# Segmentation of Gait Sequences in Sensor-Based Movement Analysis: A Comparison of Methods in Parkinson’s Disease

**DOI:** 10.3390/s18010145

**Published:** 2018-01-06

**Authors:** Nooshin Haji Ghassemi, Julius Hannink, Christine F. Martindale, Heiko Gaßner, Meinard Müller, Jochen Klucken, Björn M. Eskofier

**Affiliations:** 1 Machine Learning and Data Analytics Lab, Department of Computer Science, Friedrich-Alexander-University Erlangen-Nürnberg (FAU), Martensstraße 3, Erlangen 91058, Germany; julius.hannink@fau.de (J.H.); christine.f.martindale@fau.de (C.F.M.); bjoern.eskofier@fau.de (B.M.E.); 2 Department of Molecular Neurology, University Hospital Erlangen, Friedrich-Alexander University Erlangen-Nürnberg (FAU), Schwabachanlage 6, Erlangen 91054, Germany; heiko.gassner@uk-erlangen.de (H.G.); jochen.klucken@uk-erlangen.de (J.K.); 3 International Audio Laboratories Erlangen, Erlangen 91058, Germany; meinard.mueller@audiolabs-erlangen.de

**Keywords:** Parkinson’s disease, gait analysis, inertial sensors, step segmentation, stride segmentation, accelerometer, gyroscope

## Abstract

Robust gait segmentation is the basis for mobile gait analysis. A range of methods have been applied and evaluated for gait segmentation of healthy and pathological gait bouts. However, a unified evaluation of gait segmentation methods in Parkinson’s disease (PD) is missing. In this paper, we compare four prevalent gait segmentation methods in order to reveal their strengths and drawbacks in gait processing. We considered peak detection from event-based methods, two variations of dynamic time warping from template matching methods, and hierarchical hidden Markov models (hHMMs) from machine learning methods. To evaluate the methods, we included two supervised and instrumented gait tests that are widely used in the examination of Parkinsonian gait. In the first experiment, a sequence of strides from instructed straight walks was measured from 10 PD patients. In the second experiment, a more heterogeneous assessment paradigm was used from an additional 34 PD patients, including straight walks and turning strides as well as non-stride movements. The goal of the latter experiment was to evaluate the methods in challenging situations including turning strides and non-stride movements. Results showed no significant difference between the methods for the first scenario, in which all methods achieved an almost 100% accuracy in terms of F-score. Hence, we concluded that in the case of a predefined and homogeneous sequence of strides, all methods can be applied equally. However, in the second experiment the difference between methods became evident, with the hHMM obtaining a 96% F-score and significantly outperforming the other methods. The hHMM also proved promising in distinguishing between strides and non-stride movements, which is critical for clinical gait analysis. Our results indicate that both the instrumented test procedure and the required stride segmentation algorithm have to be selected adequately in order to support and complement classical clinical examination by sensor-based movement assessment.

## 1. Introduction

Parkinson’s disease (PD) is a neurodegenerative disorder with a prevalence of up to 2% in the elderly. The most important impairments caused by PD are bradykinesia, rigidity, tremor, and postural instability [1,2]. PD diagnosis and monitoring is mainly based on standardized scoring methods such as the Unified Parkinson’s Disease Rating Scale (UPDRS) [3] and common techniques such as gait analysis, Timed Up-and-Go (TUG) [4], and the postural stability [5] test. However, low inter-rater reliability [6] for clinical assessment has been reported, necessitating complementary approaches in the clinical setting. Besides, there has been a growing interest in the monitoring of patients outside of the clinical environment e.g., in the case of long-term monitoring. The need to fulfill these goals has led to the development of a wide range of technical systems [7,8,9].

Gait is a motor task that is progressively impaired in PD over time. Gait analysis has been performed for diagnosis, risk of fall estimation [10], quantification of quality of life [11] and many other types of assessments. There has been an upsurge in the development and validation of systems to automate gait analysis in order to assess it in a quantitative way [12,13]. The general schema of these systems includes a pipeline of gait measurement, gait processing, and gait characteristic analysis.

Gait measurement can be done with many different systems and sensors with different capabilities. Some of the most important systems being used in research are motion capture systems, which are widely accepted and provide ground truth in order to validate other systems [14,15]. Despite their high accuracy, motion capture systems are costly and can only be used within a laboratory setting. On the other hand, there exist wearable inertial sensors that have been increasingly used in gait measurement due to their light weight and capacity to be used outside of laboratories [16]. Inertial measurement units (IMUs) can consist of several sensors to measure acceleration and angular velocity. IMUs are small enough to be easily attached to different parts of the body and can be used inside and outside clinics.

Subsequent to gait measurement, the data recorded by inertial sensors are processed to obtain clinically meaningful gait characteristics. Despite diverse gait analysis applications, there has been a dominant approach to processing gait. Gait consists of periodic cycles known as strides. The first hurdle in the gait processing pipeline is to segment a gait sequence to individual strides, which is referred to as gait segmentation. In the next step, we can extract spatio-temporal parameters such as stride velocity and stride length from these individual strides [15,17,18,19] in order to analyze gait characteristics.

If gait were a completely periodic task, stride segmentation would be easy. However, in reality strides are not totally periodic and there are different sources of variation in their form, length, and amplitude. Gait disturbances vary from patient to patient (inter-patient gait variability) resulting in different stride patterns. Moreover, walking speed commonly varies with age, considerably affecting stride duration [20,21]. Strides may even vary during a short walk during a clinical test (intra-patient gait variability). For example, gait initiation and termination usually deviate from the rest of the gait sequence. These sources of variation result in a heterogeneous sequence of strides, which is one of the main challenges in stride segmentation and calls for intelligent processing methods.

Different algorithmic methods have been applied for gait segmentation, which can be summarized into three groups. The first group of methods belongs to event-based methods. Many segmentation methods have been proposed based on detection of stride events [22,23], such as toe-off and heel strike. Some of the event-based methods have used clearly defined signal characteristics like peaks [12,24,25,26], minimums, or zero crossings [27] in the gyroscope or accelerometer signal to identify events. In several works, wavelet analysis has been proposed to determine stride events. It is suggested that events are better identified in the wavelet domain instead of in the time domain [28,29,30].

An alternative to event-based methods are template matching algorithms [31,32]. This group of algorithms is used for computing the similarity between two time series. Multi-dimensional subsequence dynamic time warping (msDTW) has been used by Barth et al. [33] for gait segmentation. The method allows the identification of multiple strides in a sequence though they might differ in length, amplitude, and form.

Machine learning (ML) methods have been used successfully in many applications including the gait segmentation. Hidden Markov models (HMMs) are a widely used ML method for modeling sequences of data. Unlike the other groups of methods, HMM methods work based on representing probability distributions over sequences of strides. Several studies used hidden Markov models to segment pathological and healthy gaits [34,35,36,37]. Martindale et al. applied hierarchical HMMs (hHMMs) for gait segmentation of hereditary spastic paraplegia (HSP) patients [38].

Many methods presented have been implemented successfully for robust stride segmentation. However, not all of these studies focused on PD patients with their specific pathological gait. Furthermore, the systems used varied in terms of applied sensors and sensor placement. Moreover, their study populations differed in terms of size and characteristics. Besides this, studies reported different metrics to evaluate the segmentation methods. Due to the aforementioned reasons, a fair comparison of the gait segmentation methods is currently impossible.

Hence, the goal of this work is to contribute a comprehensive comparison of four prevalent gait segmentation methods for PD. These are peak detection, two variants of DTW methods (Euclidean DTW (eDTW) and probabilistic DTW (pDTW)), and hHHM [12,33,34]. We examined two experiments with different levels of complexity that represented a wide range of gait studies in PD [15,39,40]. Through these two experiments, we analyze the advantages and disadvantages of each method for sensor-based movement analysis in PD. In particular, our comparison of methods reveals which method works the best for each assessment paradigm and can be applied in similar cases. We further discuss avenues for future work.

## 2. Methods

In this section, we present a general overview of the methods applied. In this paper, we aim only to highlight the main differences between these groups of methods; references have been provided for more detailed presentations of each method.

### 2.1. Peak Detection

Identifying peaks in a given data sequence is important in many applications, as they often indicate significant events in the signal. Formulation of a peak detection method depends on the specific signal characteristics. However, usually two basic requirements must be fulfilled to identify a data point as a peak. First of all, the signal magnitude should be higher than a certain threshold, which can be set based on the signal characteristics. Moreover, the minimum time between two consecutive peaks must be greater than a certain threshold to avoid finding two or more peaks in one stride. Other requirements can be applied as well, e.g., the first and second derivatives of the signal may meet certain criteria. Performing these straightforward steps, we can segment the stride using the identified peaks.

### 2.2. Multi-subsequence Dynamic Time Warping

In general, DTW is used to find the similarity between two time-series sequences. msDTW is an extension of DTW with the goal of finding multiple subsequences in a larger sequence, each being similar to a given shorter sequence [31,33]. To segment a sequence into strides, we constructed a template and tried to find multiple subsequences in the sequence, each being similar to the template. The algorithm of msDTW is as follows:

The template is modeled as a sequence $X=(x_{1},x_{2},\cdots ,x_{M})$ of length *M* with elements $x_{m}$ for m∈{1,⋯,M}. Similarly, the gait sequence for our observation is modeled as $Y=(y_{1},y_{2},\cdots ,y_{T})$ having a length *T* with elements $y_{t}$ for t∈{1,⋯,T}. The length *T* of *Y* is much larger than the length *M* of *X*.

1. Distance matrix D: The elements of D represent the pairwise distance between the elements of the template *X* and the gait sequence *Y*. The size of the matrix D is M×T. In the case of including several axes, separate distance matrices are computed and they are all summed up to construct a single distance matrix [33].
2. Accumulated cost matrix C: represents the distance between the template and the gait sequence as well as the accumulated costs of warping the template to parts of the gait sequence. The bottom row of matrix C is as follows:
  C(1,t)=D(1,t)∀t∈{1,⋯,T}
  The first column is:
  $$C\left(m,1\right)=\sum_{i=1}^{M}D\left(i,1\right)\;\;\forall m\in \left\{1,\cdots ,M\right\}$$
  The remaining elements are calculated in a recursive manner as
  $$\begin{array}{c} C(m,t)=D(m,t)+min\{C(m-1,t-1),C(m-1,t),C(m,t-1)\}\\ \forall m\in \{1,\cdots ,M\},t\in \{1,\cdots ,T\} \end{array}$$
3. Distance function Δ: The top row of matrix C represents the accumulated costs for warping the stride template *X* to the gait sequence *Y* and can be considered as a matching function Δ:[1:T]→R.
4. Warping path *P*: Warping path $P=(p_{1},p_{2},\cdots ,p_{L})$ of length *L* with elements $p_{l}$ for l∈{1,⋯,L} presents a good match between *X* and *Y*. Local minimums of the matching function Δ are considered as the end points of warping paths and starting points are obtained by backtracking on the accumulated cost matrix. A threshold should be chosen in order to select these local minimums in such a way to find the maximum number of relevant strides in the sequence.
5. Boundary conditions for a complete stride:
  - Start of warping path *P* is in the top row of the cost matrix C.
  - End of warping path *P* is in the bottom row of cost matrix C.
  - Condition to ensure warping path monotonically decreases:
    $$p_{l+1}-p_{l}\in \left\{\left(1,0\right),\left(0,1\right),\left(1,1\right)\right\}\;\;for\;\;l\in \left\{1,\cdots ,L\right\}$$

Different variants of DTW method differ in the cost function used to compute the distance matrix D and template generation. In this work, we used Euclidean and probabilistic-based cost functions.

For generating a template, a set of strides of any form and size is considered. For eDTW, the strides are interpolated to the size of the template *M* and are averaged sample by sample to generate a representative template. Then, in the first step of the DTW algorithm, the Euclidean distance between the samples of the average template *X* and the gait sequence *Y* is computed.

In the case of pDTW [41], a probabilistic template is constructed. Once all training strides are aligned to the same length *M*, the sample vector at a certain point *m* among all strides is modeled by means of a Gaussian distribution. As a result, *M* Gaussian distributions construct a probabilistic template with the length *M*. To find the distance of a gait sequence to this probabilistic template, we compute the probability P(y) of a given sample *y* belonging to these Gaussian distributions. These probabilities provide a similarity measure between samples of the gait sequence *Y* and elements of the template *X*. To turn the similarity measure to the required distance measure D(.), we use an exponential-based measure of the probability:

D(y)=exp(−P(y))

### 2.3. Hierarchical Hidden Markov Models

Hidden Markov models are probabilistic frameworks for sequential data analysis [42,43], which are used in many application domains [44,45]. In this paper, we use a variation of the HMM called the hierarchical HMM (hHMM) [46], which is different from conventional HMMs mainly in the structure of the model. In the hHMM, it is possible to define a hierarchy of model states, which makes it more suitable for gait segmentation.

With the standard HMM, a sequence of observations is represented using probabilistic distributions. In this application, observations are gait data. Let us denote the observation at time *t* by the variable $y_{t}$. We assume the observation at time *t* is generated by some process whose states $s_{t}$ are hidden. The states of this hidden process satisfy the Markov property, which means given the value of hidden state $s_{t-1}$ the current state $s_{t}$ is independent of all the states prior to t−1. To define a probability distribution over observations, we need the initial probability over hidden states $P(s_{1})$, the state transition matrix defining $P(s_{t}|s_{t-1})$, and the observation model defining $P(y_{t}|s_{t})$. In this work, observations are modeled by Gaussian mixture models (GMMs).

**Figure 1:**
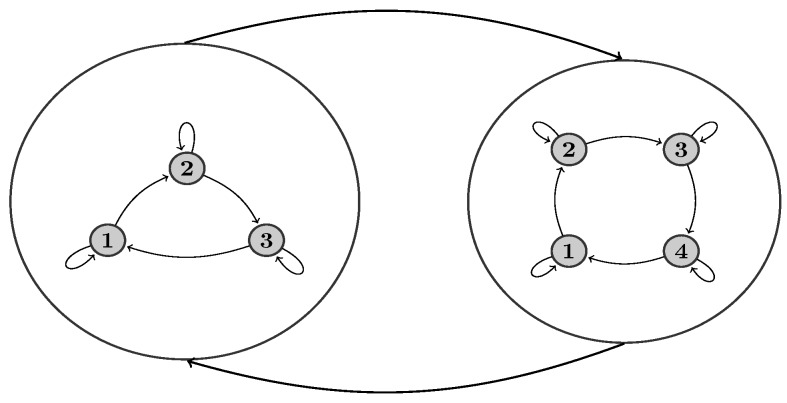
Topology of a two-level hierarchical hidden Markov model (hHMM). Large circles represent states in the first level of the model. Each state in turn is an HMM with sub-states (dark circles). Here, we applied left-to-right HMMs in the second level.

From a topological point of view, hHMMs [46] generalize the HMMs by making each of the hidden states a probabilistic model on its own. That is, each state is an HMM in the case of a two-level hierarchy (see Figure 1). The HMMs in the second level have states in turn that are referred to as sub-states. Transitions can be taken place between states in one level or between states and sub-states in different levels. The lowest level sub-states define the observation model $P(y_{t}|s_{t})$.

Learning in hHMM entails estimation of the parameters of the hHMM, including transition and initial probabilities and GMM parameters based on given data. After learning a model, we can perform inference, which in our application means finding the most probable sequence of states $S^{*}$ given an observation sequence with the size *T*:

(1) $$S^{*}=\underset{S_{1:T}}{\mathrm{argmax}}P\left(S_{1:T}|Y_{1:T}\right)$$

## 3. Evaluation Study

We apply four methods, namely peak detection, eDTW, pDTW, and hHMM to the problem of gait segmentation from foot-worn IMUs. Peak detection, msDTW, and hHMM are widely used for gait segmentation. pDTW has been used in other applications such as gesture recognition [41]. To the best of our knowledge, pDTW has not been applied to gait segmentation before. It is worth mentioning that while the implementation procedures presented here can be replicated for similar cases, the examined range of parameters that will be presented in this section highly depends on the data set at hand.

### 3.1. Data Collection and Setup

Ten patients diagnosed with idiopathic PD (63 ± 9.3 years old, 5 males) with a UPDRS motor score of 12.7 ± 6.0 and Hoehn and Yahr (H&Y) score of 1.7 ± 0.9 were included in the first experiment. For this experiment, patients walked 10 m four times at a self-selected speed. Between each 10-m walk, there was a 180° turn, which was excluded from the data using videos. Hence, the final data included only a sequence of straight walk strides. For this experiment, the total number of strides for all patients was 496.

For the second experiment, the population consisted of 34 patients with idiopathic PD (63 ± 11 years old, 24 males). Subjects were in early to moderate stages of the disease with a UPDRS motor score of 18.8 ± 8.9 and H&Y score of 2.2 ± 0.6. The total number of strides for this experiment was 458. Each patient performed a TUG test at a self-selected speed. The TUG test is a commonly used clinical test to evaluate balance and mobility. The patient stands up from a chair, walks for 3 m, performs a 180° turn, walks back for 3 m and finally sits again [4]. The test includes straight walking and turning. In PD, turning is more impaired than straight walk [47], and hence, data from this experiment have a higher intra-patient gait variability and result in a more heterogeneous set of strides than the first experiment. Transitions between sit-to-stand and stand-to-sit make stride segmentation challenging, because it is essential for the methods to distinguish transition movements from stride movements. All patients were capable of finishing the TUG test without episodes of freezing or dyskinesia. For both experiments, patients gave written informed consent approved by the local committee of the medical faculty at University of Erlangen, Germany (Re.-No. 4208), which follows the declaration of Helsinki 1975, as revised in 2000.

**Figure 2:**
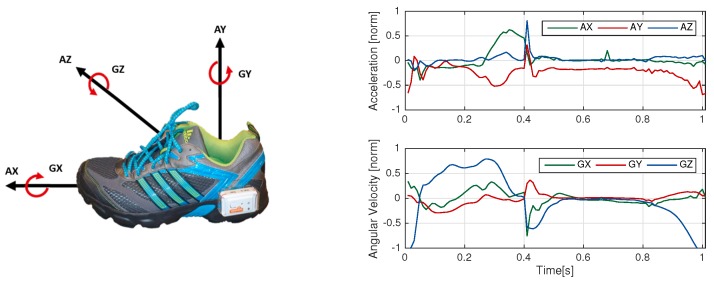
(**Left**) Shimmer sensor placement and axes definition (AX, AY and AZ form three dimensions of accelerometer and GX, GY and GZ form three dimensions of gyroscope); (**Right**) Accelerometer and gyroscope data for one exemplary stride.

For both experiments, data was recorded by a Shimmer 2R (Shimmer Sensing, Dublin, Ireland) IMU, recording acceleration and angular velocity at 102.4 Hz. Each unit consisted of a tri-axial accelerometer (range ± 6 g) and a tri-axial gyroscope (range ± 500 °/s). The sensor units were mounted laterally to the ankle of the patient’s right and left shoes. The measurements from both feet were included in the experiments. Figure 2 shows the sensor placement on the shoe and the axes definition as well as sample data for one stride normalized to the range of the sensors (norm).

### 3.2. Manual Data Labeling

**Figure 3:**
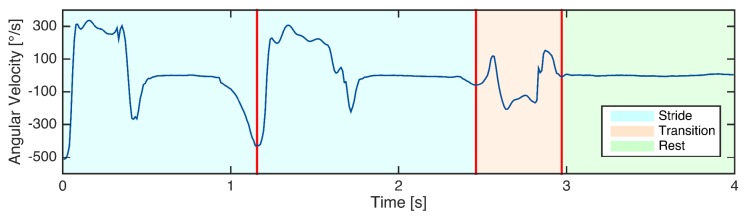
Labeling of an example of gyroscope signal including two strides, the following transition (non-stride) movement, and rest.

The strides were labeled using simultaneous analysis of video and sensor data. The video and sensors were synchronized using a synchronization movement based on lifting one foot three times. The start and end point of each stride was labeled manually using acquired information of gyroscope and the stride definition from [33]. Angular velocity in the sagittal plane (GZ) was used. The negative peaks in GZ represent the change in foot rotation during one stride and were used to define start and end of the strides. Stride start was set to the negative peak before swing phase and stride end to the negative peak at the end of the stance phase (see Figure 3). Videos were used to accurately identify the negative peaks. In order to map each video frame to a sample in the GZ signal, a toolbox was used, which was implemented for this purpose. The end of one stride coincides with the start of the following stride for consecutive strides.

For the TUG segmentation, in addition to strides, rests and transitions were labeled. The rest phase refers to the part where patient stands still and transition is any movement other than stride movements as defined by [38]. Figure 3 shows an example of the way the gait sequence was labeled. The labeling was performed by a person familiar with gait data.

### 3.3. Implementation of Peak Detection

For peak detection the gyroscope signal Z-axis (GZ) (See Figure 2) was used [15,33]. Peaks in the GZ signal corresponded to the middle of swing phase in the strides. For this method, only one point in the stride and no stride borders were recognized. There were two conditions in order to detect a peak. Firstly, angular velocity must be greater than 150 °/s [15,33]. Moreover, the time to previous and following peaks must be greater than 600 ms, which was considered as the lower bound for length of a stride. This time constraint was applied equally for all methods. In the case of detecting multiple peaks in this region, only the highest amplitude was selected. For implementation, the peak detection function in MATLAB 2015a was used.

### 3.4. Implementation of Euclidean DTW

The input to the DTW was raw data [33,38]. For template generation, we chose a template of the size 200 samples (M=200). Template must have a proper length to capture subtle variations in strides. Manually segmented strides were linearly interpolated to the size of 200 samples and the average of a sample vector at a certain point *m* among all strides was computed. The template signals were then normalized to the range of sensors (±6 g for accelerometer and ±500 °/s for gyroscope axes).

**Figure 4:**
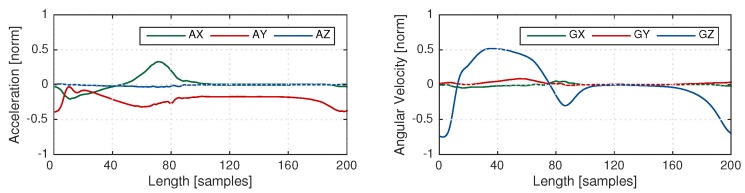
Template based on average of (**Left**) a three-dimensional accelerometer signal, (**Right**) a three-dimensional gyroscope signal. Signals AX, GX, and GY have a very low variation.

Figure 4 shows the signals of the template for eDTW. The signals AZ, GX, and GY are nearly constant and do not convey information. Hence, three signals of AZ, GX, and GY (See Figure 2) were omitted from computations. The combination of signals and threshold used for template matching is shown in Table 1. As mentioned in Section 2.2, thresholds in the DTW algorithm were used to determine the end boundary of the strides, which was in turn based on the distance between the template and part of the gait sequence. Using multiple axes instead of one axis increased the distance, and therefore, the threshold was increased accordingly (see Table 1). In addition, the time of an overlap of a given warping path must be less than 200 ms for the stride to be segmented [33]. In a post-processing step, time constraints were applied to the output of the algorithm. A stride must be larger than 600 and smaller than 2500 ms [33]. These time constraints were equally applied for pDTW and hHHM algorithms. Template generation and eDTW algorithms were implemented in MATLAB 2015a.

### 3.5. Implementation of Probabilistic DTW

The input to pDTW is raw data and the same constraints as used in eDTW were applied here as well. The template generation and computing distance between the gait sequence and template are explained in Section 2.2.

The output of a probability density function is between 0 and 1 for univariate and multivariate data. Hence, the output of the distance function is the same for single-axis (univariate) or multi-axes (multivariate) data. Accordingly, the threshold stayed the same for any combination of axes. Due to the difference between Euclidean and probabilistic cost functions, range of thresholds for eDTW and pDTW algorithms are different. Table 2 shows the combination of the axes and thresholds. Again, template generation and pDTW algorithms were implemented in MATLAB 2015a.

### 3.6. Implementation of hHMM

A two-level hHMM was considered for gait segmentation for both experiments. In the first experiment, there was only one state to capture strides, while in the second experiment, there were three states of stride, rest, and transition. The second level of hHMM included left-to-right HMMs, which could include multiple sub-states in turn. The exact number of sub-states was determined by optimization. It is expected that the number of required sub-states grows as the pattern becomes more complex. In the first level, learning was done in a supervised manner using labels of stride borders, while in the second level, an unsupervised approach was applied. The advantage of semi-supervised learning is that we do not need to provide labels for the second level, but learning is done based on the underlying data.

The input to the hHMM was a set of features extracted from the raw data using the sliding window approach. In this approach, the data was segmented into overlapping time frames. The windowing was done using the Hann window instead of rectangular window in order to reduce the effect of windowing on the edges. From each window a set of features was extracted, including the raw data itself, mean, variance, energy and three coefficient of the second order polynomial fit [38]. The final feature set was constructed by concatenating the features from all IMU axes. The feature set was then normalized. The size of the sliding window was chosen in a way that the features best represent the underlying data. Several window sizes were tried as in [36,38]. To get the most relevant features and reduce the dimensionality of data (and therefore number of parameters), we used principal component analysis (PCA).

For optimizing the number of principal components that was fed to the hHMM, as well as parameters that controlled the structure of the hHMM (such as number of sub-states and number of components per GMM), a grid search was used. Table 3 shows the values for these parameters, which were chosen partially based on literature [36,38] and partially empirically. hHMM model parameters included transition matrices and initial state probabilities as well as GMM parameters, including means, diagonal covariance matrices, and weights of GMM components. The first-level transition matrix and GMM parameters were initialized based on the data distribution. Transition matrices for second-level HMMs were initialized uniformly. For learning model parameters, the Baum–Welch (BW) [48] algorithm was applied, which is a special case of the expectation maximization (EM) algorithm [49]. The BW algorithm was performed at most for 20 iterations. For inference and gait segmentation the Viterbi algorithm [50] was used.

For feature extraction and dimensionality reduction, MATLAB 2015a was used because it provided all the necessary functions. For learning and inference of the hHMM the Java Speech Toolkit (JSTK) was used [51], since this toolbox allows for semi-supervised learning and inference.

### 3.7. Performance Assessment

The goal in segmentation was two-fold: (1) to minimize the number of missed strides; and (2) to minimize signal parts which are wrongly detected as strides. True positives (TPs) are strides segmented by the method and are also labeled as strides in the ground truth. False negatives (FNs) are the strides that are not recognized by the segmentation algorithms. If there is no ground truth stride and a method segments a stride, for example, at rest or in transition time, then a false positive (FP) occurs. Based on the mentioned parameters, three metrics are computed. Precision considers false positives and is equal to one only if all the recognized strides are labeled in the ground truth. Recall considers the false negatives and is equal to one if no stride is missed. The F-score, which takes into account missing strides and wrongly detected strides equally, is the main metric for comparison of methods and grid search optimization has been performed based on that.

(2) $$\mathrm{Precision}=\frac{\Sigma TP}{\Sigma TP+\Sigma FP}$$

(3) $$\mathrm{Recall}=\frac{\Sigma TP}{\Sigma TP+\Sigma FN}$$

(4) $$F\text{-}\mathrm{score}=2\times \frac{\mathrm{Precision}\times \mathrm{Recall}}{\mathrm{Precision}+\mathrm{Recall}}$$

For all methods the segmented strides were compared with the ground truth stride borders and were marked as correctly segmented if the start and end borders were within ±100 ms of the ground truth borders, which is approximately 10% of stride time [33,37,52].

## 4. Experimental Results

The first experiment was performed in a leave-one-out cross validation scheme. Data from both feet of one patient were left out on each iteration and the rest of the data was used as a training set. Parameter tuning as well as template generation were performed based on the training set. Three of the methods of choice (hHMM, eDTW and peak detection) could detect all strides with a F-score of 100%. Probabilistic DTW yielded a slightly worse result, with the F-score of 99.8 ± 0.4%.

In the case of the second experiment, due to the larger data set, a 4-fold outer cross-validation was applied for the evaluation of methods. For validation and parameter estimation, an inner 4-fold cross validation was used. The cross-validation was performed such that no patient used for training and validation appeared in the test set. To remove any possible bias, the data was randomized for choosing the test and validation sets. The randomization was equally applied for all methods. Table 4 lists average statistics across test folds for the best set of parameters in each method. Methods were evaluated based on their F-scores.

**Figure 5:**
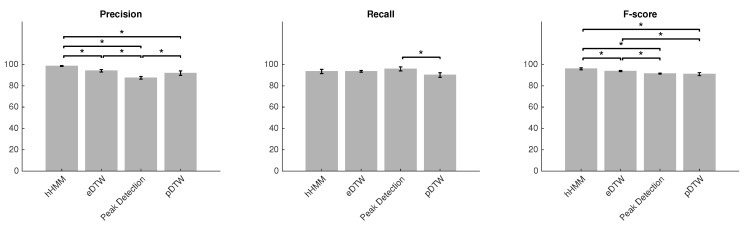
Mean ± STD of precision, recall and F-score for four methods. Asterisks represent a 5% significant difference between methods corresponding to 95% confidence interval.

To identify significant differences between methods, statistical tests were performed. The Wilcoxon test was used as a non-parametric statistical test for pair-wise comparison of the result because of the small number of samples and possibility of having non-normal distributions. Figure 5 shows the result of the pair-wise tests. In the case of precision, all tests showed a significant difference (p < 0.05) except for the comparison of eDTW to pDTW (p = 0.20). The result showed higher variance for the recall metric. The methods showed no significant difference (p > 0.05) in the case of recall excluding the test between peak detection and pDTW (p < 0.05). For the F-scores, the variance for all methods decreased, which accounted for the significant difference in most of the tests (p < 0.05) except for the test between peak detection and pDTW (p = 0.88).

## 5. Discussion and Conclusion

One main approach to the quantitative assessment of gait in PD is to analyze spatio-temporal parameters extracted from individual gait strides [53,54], which highlights the importance of robust stride segmentation. We compared four prominent segmentation methods with the focus on pathological gait of PD patients. In order to cover wide range of gait studies in PD [15,39,40], we assessed gait segmentation methods under two scenarios with different levels of complexity. In the first scenario, a data set including only straight walk was considered. The second scenario focused on a more challenging data set including stride and non-stride movements, as well as turnings. Intra-patient gait variability increased as turning strides were combined with strides from straight walking.

The result from the first scenario showed the methods perform similarly well, with 100% accuracy. This result suggested that when there is only a sequence of strides with low variability derived from a very rigid supervised test assessment paradigm, all methods perform similarly. In such cases, one may consider using a simpler and faster method, especially for large data sets. The peak detection method does not need parameter learning and is the fastest method. On the other hand, there are HMM methods for which the parameter learning phase can be computationally costly. However, once the model is learned, it can be used for further gait segmentation either in an offline or online mode.

In contrast, in the second scenario, the performance of all methods diminished considerably. The methods also demonstrated different performances (see Table 4). The hHMM significantly outperformed the other methods with an accuracy of 96% and a low standard deviation, which is a promising result for gait analysis applications. eDTW yielded a 94% F-score while peak detection and pDTW obtained only a 91% and 90% F-score, respectively.

Gait analysis systems using wearable inertial sensors have made long-term monitoring of PD patients possible. Different studies were conducted to monitor and analyze gait fluctuations in PD during the course of a day [55,56]. The most important aspect of a gait segmentation method for long-term monitoring lies in its ability to deal with gait variability in a non-supervised and non-standardized test setting with a high accuracy. Our experiments demonstrate how the methods can deal with variability, though on a smaller scale than in long-term monitoring.

The power of ML methods is increasingly appreciated in PD studies [18,40]. They also prove promising in the case of sensor-based gait segmentation [35,38]. Our results revealed that for dealing with inter- and intra-patient gait variability, hHMM methods surpassed the other methods. The hHMM achieved a high precision of nearly 99%, meaning that there was a low rate of false positives. The probabilistic representation of the data was effective in distinguishing between stride and non-stride movements and there were only a few cases in which non-stride movements were segmented as strides. The other group of ML methods that can be used for stride segmentation are deep learning (DL) methods [18,57], which in the emergence of high computational power and large data sets become increasingly popular. DL methods have advantages over HMM methods, since they perform feature extraction automatically. However, learning their large parameter space requires availability of a large data set. Size of our data set ruled out the possibility of applying these methods.

eDTW significantly surpassed pDTW by 4% in terms of the F-score. The templates in eDTW were generated simply by computing the average of strides, while in pDTW a series of probability distributions modeled the template. It was speculated that a probabilistic template would result in a more flexible template than an average-based template. However, in practice, eDTW proved more effective in gait segmentation. It is worth noting that pDTW is a probabilistic template matching method and does not utilize the fully probabilistic representation of data the same way as HMM methods do.

Peak detection yielded the best recall of 95% among all methods in the second experiment. From a methodological point of view, peak detection is a very simple method in which, unlike hHHM and DTW methods, there is no need for parameter learning. In particular, peak detection is a good method of choice in case of small size data sets, where enough data is not available for parameter tuning. However, the low precision rate in the case of the second experiment suggests that the applicability of this method is limited in case of more complicated data sets, since it produces many false positives.

Although the F-score was the main metric for performance evaluation, in clinical applications of gait analysis, a low false positive rate is more critical than a low false negative rate. This is because clinical gait analysis, which follows gait segmentation, is based on statistics of spatio-temporal parameters extracted from strides. Parameters extracted from false positive strides may destroy the underlying statistics. Hence, in PD studies the precision is more critical than the recall. Precision versus recall tendencies vary among methods. DTW methods showed a balance between precision and recall, while hHMM yielded a high precision and peak detection a high recall. Precision in hHMM is significantly higher than all other methods.

The main limitation of the methods stemmed from their low recall rate. Statistical analysis showed no significant difference between recall rates, except for the comparison between pDTW and peak detection. The methods segmented all strides correctly in a homogeneous sequence as shown by the first experiment. However, in a heterogeneous setting, (as shown by our second experiment), all methods tended to miss strides that deviate from normal strides in form and length. In such cases, hHMM might fail to generalize to these strides and the template in DTW methods might not be able to match such strides. One solution for that is to have a large enough number of such atypical strides in the data set. Although we used one of the largest data sets for the gait segmentation problem in the literature [34,38], an even larger population may mitigate the problem of variable strides. It is a general rule in any application that a large population can lead to a better representation of data in HMM and in the same manner more generic templates for DTW methods.

The inter-patient gait variability may be more effectively addressed using individualized models, in such a way that models better reflect the specific gait charactristics of each individual patient. In particular, as the PD progresses, the motor impairment deteriorates, which results in a larger deviation of pathological gait from normal gait. An atypical gait pattern that largely deviates from the average of the population results in a poor performance of the methods. In such extreme cases individualization can be helpful. hHMM provides the theoretical foundation to adapt models to individual patients [58]. For DTW methods, it is also possible to construct templates based on an individual patient. An individualized template may map the patient’s strides better than a generic template.

Lastly, gait analysis can provide valuable clinical information also for other neurological disorders that affect gait such as HSP [38] or multiple sclerosis. Gait disturbances vary among these diseases, and hence, segmentation methods should be adapted to specific gait patterns of each disease.

In summary, automated mobile gait analysis offers an elaborate assessment of pathological gait, leading to a deeper insight into PD. To assess sensor-based gait segmentation, which is an important building block in the process of gait analysis, we compared four segmentation methods widely used in the literature. The experiments showed the accuracy of segmentation methods to a great extent depends on the stride variability in data sets that is mainly derived from the variations of the instrumented test paradigm, the pathological gait of PD patients, the specific gait patterns of each patient, and the difference between straight and turning movements. In the case of a homogeneous data, even a simple method such as peak detection proved effective, while, in the case of more heterogeneous assessment paradigms reflecting the standardized test paradigms along with non-supervised and non-standardized assessments e.g. in long-term monitoring, probabilistic hHMM significantly outperformed the other methods. The results of the current study can be applied to any PD studies inside the clinic and provide useful insights for long-term monitoring outside the clinic.

## Figures and Tables

**Table 1:** Euclidean dynamic time warping (eDTW) parameters.

Signal Combination	GZ	AXGZ	AYGZ	AXAYGZ
Threshold (steps of 5)	10–25	20–30	20–30	25–40

**Table 2:** Probabilistic DTW (pDTW) parameters.

Signal Combination	GZ	AXGZ	AYGZ	AXAYGZ
Threshold (steps of 1)	8–15	8–15	8–15	8–15

**Table 3:** hHMM parameters.

Parameters	Values
Sliding window length (s) (steps of 0.20)	0.10–0.70
Number of sub-states for stride | transition | rest (steps of 2)	4–12 | 2–4 | 1
Number of Gaussian mixture model (GMM) components (steps of 2)	8–12
Number of principal components (steps of 2)	1–15

**Table 4:** Results of the second experiment in terms of precision, recall, and F-score.

Method	Precision (%)	Recall (%)	F-score (%)
hHMM	98.5 ± 0.4	93.5 ± 1.9	95.9 ± 0.9
eDTW	94 ± 1.2	93.5 ± 0.8	93.8 ± 0.5
Peak detection	87.4 ± 1.2	95.9 ± 1.8	91.5 ± 0.4
pDTW	91.8 ± 2.1	90.1 ± 2.2	90.9 ± 1.4
